# Novel pathogenic mutations identified in the first Chinese pedigree of complete C6 deficiency

**DOI:** 10.1002/cti2.1148

**Published:** 2020-07-08

**Authors:** Philip H Li, William WY Wong, Evelyn NY Leung, Chak‐sing Lau, Elaine Au

**Affiliations:** ^1^ Division of Rheumatology & Clinical Immunology Department of Medicine Queen Mary Hospital The University of Hong Kong Hong Kong; ^2^ Division of Clinical Immunology Department of Pathology Queen Mary Hospital Hong Kong

**Keywords:** C6, Chinese, complement, deficiency, immunodeficiency, Neisseria

## Abstract

**Objectives:**

Complete C6 deficiency (C6Q0) is a rare primary immunodeficiency leading to increased susceptibility to recurrent *Neisseria* infections. Patients with C6Q0 have mostly been reported in individuals of African ancestry previously, but never in Chinese. We identify the first Chinese patients with C6Q0 through family screening of an index case presenting with recurrent *Neisseria* meningitis with septicaemia and performed extensive clinical, serological and genetic investigations.

**Methods:**

Two variants in *C6* were identified by next‐generation sequencing and confirmed by Sanger sequencing in an index case of C6Q0. Immunological investigations, complement haemolytic assays (CH50/AH50), *C6* gene sequencing and quantification of serum C6 levels were performed for all available members of his nonconsanguineous family.

**Results:**

Three C6Q0 patients were identified with near‐absent C6 levels, absent CH50/AH50 activity and compound heterozygous for two nonsense mutations in the *C6* gene: NM_000065.4:c.1786C>T (p.Arg596Ter) and NM_000065.4:c.1816C>T (p.Arg606Ter). Neither mutations have been reported to be pathogenic previously. Two other family members who were heterozygous for either p.Arg596Ter or and p.Arg606Ter had intermediate C6 levels but preserved CH50/AH50 activity. These two loss‐of‐function mutations showed a strong genotype–phenotype correlation in C6 levels.

**Conclusions:**

We report on two compound heterozygous mutations in *C6*, p.Arg596Ter and p.Arg606Ter inherited in three patients of the first recorded Chinese pedigree of C6Q0. Neither mutations had been reported to be pathogenic previously. We demonstrate that heterozygous family members with subtotal C6 levels had preserved complement haemolytic function and demonstrate a threshold effect of C6 protein level.

## Graphical abstract

We report on two compound heterozygous mutations in C6 (p.Arg596Ter and p.Arg606Ter) in the first recorded Chinese pedigree of complete C6 deficiency. We demonstrate that heterozygous family members with subtotal C6 levels had preserved complement hemolytic function and demonstrate a threshold effect of C6 protein level.

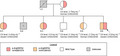

## Introduction

Human complement component C6 is one of the essential terminal complement components (TCC) and is crucial for assembly of the membrane attack complex responsible for osmotic lysis of susceptible cells.^1^ Patients with C6 deficiency (OMIM: 612446) are categorised functionally into subtotal (C6SD) or complete [also known as quantitatively zero (C6Q0)].^2^ Similar to other TCC deficiencies, C6Q0 is a primary immunodeficiency (PID) characterised by increased susceptibility to *Neisseria* spp.^1^, ^3^, ^4^, ^5^ *C6* is a single‐copy gene located on chromosome 5p13 and contains 18 exons.^6^ C6Q0 is an autosomal recessive PID, and patients suffer from significantly more serious illness or death as sequelae from recurrent meningococcal episodes.^7^ Conversely, as < 10% of normal C6 is required for sufficient complement activity, C6SD is not associated with meningococcal disease and has even been postulated to possibly confer evolutionary benefit by reduced antimicrobial activity but less severe endotoxin shock.^8^, ^9^

C6Q0 has been reported mostly in individuals of African ancestry with an estimated prevalence of 1 in 1900 in the Western Cape of South Africa.^7^, ^10^, ^11^, ^12^ Seemingly rarer, there have also been reports of C6Q0 Caucasian patients.^13^, ^14^, ^15^, ^16^ However, unlike C9 deficiency (the most common TCC deficiency in the oriental populations), C6 deficiencies have seldomly been reported in Asians, especially in Han Chinese.^17^ We identified the first Chinese patients with C6Q0 through family screening of an index case presenting with recurrent *Neisseria* meningitis with septicaemia. Extensive clinical, serological and genetic investigations were performed. We describe two compound heterozygous mutations in *C6*, p.Arg596Ter and p.Arg606Ter (neither reported to be pathogenic previously), inherited in three patients of the first reported Chinese pedigree of C6Q0.

## Results

### Genetics analysis of the C6 gene in the index and family

**Figure 1:**
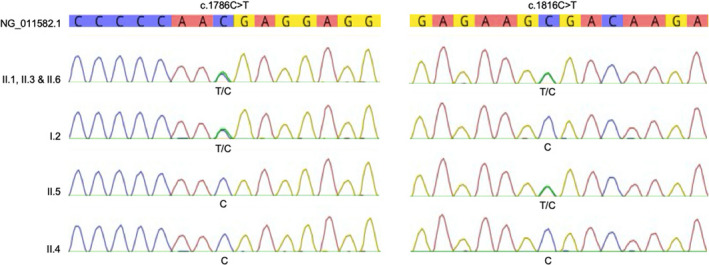
Sequence variations were identified in the *C6* gene.

**Figure 2:**
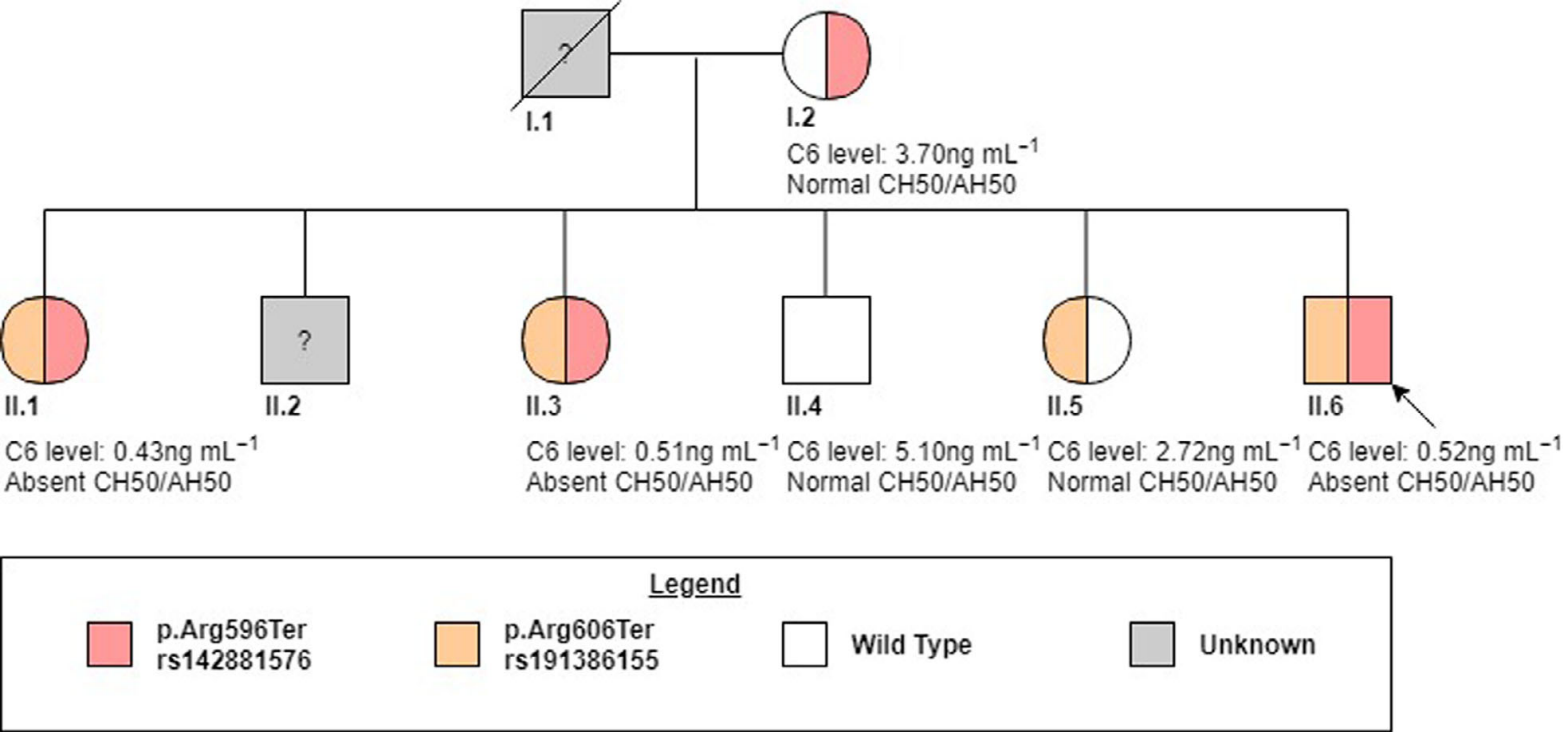
Family tree and summary of C6 genotypes.

Genotyping of *C5* to *C9* genes of the indexed patient was performed by next‐generation sequencing (NGS). Mean coverage for the targeted genes was at least 200× (data not shown). NGS revealed two nonsense variants in the *C6* gene: NM_000065.4:c.1786C>T (p.Arg596Ter) and NM_000065.4:c.1816C>T (p.Arg606Ter). These two variants were confirmed by Sanger sequencing. Detailed sequence variations in the *C6* gene of the patient and his family members are shown in Figure 1. Summary of the pedigree and mutations is shown in Figure 2. According to the public databases (i.e. Human Gene Mutation Database, dbSNP, NHLBI Exome Sequencing Project, Ensembl, 1000 Genomes Project, ExAC, GnomAD, ClinVar), p.Arg596Ter (rs142881576) was previously reported to be *likely pathogenic*. The variant results in a premature termination codon that causes the truncation of a 934‐amino acid protein into a 596‐amino acid protein. This variant has been reported with a very low frequency (A = 0.00014; 36/250704, GnomAD).^18^, ^19^ Similarly, p.Arg606Ter (rs191386155) leads to a premature termination codon and also reported with extremely low frequency (A = 0.00002; 5/250868, GnomAD).^18^ Based on our data and following the ACMG 2015 guidelines on the interpretation of sequence variants, we graded the above two variants pathogenic (i.e. 1 very strong: PVS1; AND ≥ 2 moderate: PM2, PM3).^20^

The index patient's mother (I.2) was found to be a heterozygous carrier of p.Arg596Ter. Although the patient's father (I.1) was not available for testing, the patient's fifth sibling (II.5) was noted to be a heterozygous carrier of the other mutation p.Arg606Ter. Based on this genetic pedigree, the two variants were identified in the index patient (II.6) located on separate DNA strands (i.e. *trans* phase) and the patient was a compound heterozygote. Of the remaining three siblings, two (II.1 and II.3) were also compound heterozygous for p.Arg596Ter and p.Arg606Ter and one (II.4) was wild type for both alleles.

### Quantification of C6 and haemolysis assays (CH50/AH50)

These two loss‐of‐function mutations showed a strong genotype–phenotype correlation in C6 levels and summarised in Table 1. Enzyme‐linked immunosorbent assay (ELISA) for C6 levels was performed in 8 healthy individuals, with a mean (range) level of 4.3–7.3 ng mL^−1^.

**Table 1:** Genotype–phenotype correlation of compound and single heterozygous C6 mutations

	Patient		Genotype	Phenotype	
				C6 level (ng mL^−1^)^a^	CH50/AH50
Compound heterozygous	II.1	F/35	p.Arg596Ter/p.Arg606Ter	0.43	Absent
	II.3	F/29	p.Arg596Ter/p.Arg606Ter	0.51	Absent
	II.6	M/23	p.Arg596Ter/p.Arg606Ter	0.52	Absent
Single heterozygous	I.2	F/62	p.Arg596Ter/WT	3.70	Normal
	II.5	F/26	p.Arg606Ter/WT	2.72	Normal
Wild type	II.4	M/28	WT/WT	5.10	Normal

WT, wild type.

a C6 levels also performed in eight healthy individuals by our lab, with a mean (range) level of 4.3–7.3 ng mL^−1^.

Normal controls and wild‐type sibling (II.4) had normal C6 levels of > 5 ng mL^−1^ and normal CH50/AH50. All three compound heterozygotes (II.1, II.3, II.6) of p.Arg596Ter and p.Arg606Ter had near‐absent (<1 ng mL^−1^, i.e. C6Q0) and absent CH50/AH50. The mother (I.2) and fifth sibling (II.5) with one heterozygous mutation of p.Arg596Ter and p.Arg606Ter, respectively, had C6 levels intermediate of wild type and C6Q0 (2.72–3.70 ng mL^−1^, i.e. C6SD), but preserved CH50/AH50 activity.

## Discussion

We describe the first Chinese pedigree of C6Q0 with two mutations, p.Arg596Ter and p.Arg606Ter, neither of which had been reported to be pathogenic previously. The mutations occurred in exons 12 and 13 leading to premature termination codons at positions 596 and 606, respectively. Hence, these mutations are expected to cause nonsense‐mediated decay of the altered transcripts and absence of gene product. Both nonsense mutations likely lead to abolishment of C6 protein level and function, which are reflected by a strong genotype–phenotype correlation in C6 levels.

In this family, compound heterozygosity of C6 resulted in C6Q0 while single heterozygosity resulted in C6SD. As similarly described in other mutations, C6SD does not lead to any significant clinical phenotype or susceptibility to *Neisseria* infections.^10^ In this study, we demonstrate that even about half the level of normal C6 levels was sufficient to maintain normal haemolytic activity as demonstrated by normal CH50/AH50 assays (i.e. a threshold effect of C6 protein level). Despite never yet experienced meningococcal disease previously, two compound heterozygous siblings (II.1 and II.3) with C6Q0 were identified who had not yet experienced meningococcal disease. Given the severity of the index patient's (II.6) previous infections and complete absence of CH50/AH50 haemolytic activity, all C6Q0 patients were administered complete courses of meningococcal serotype B and ACYW vaccinations and offered long‐term antibiotic prophylaxis.

To the best of our knowledge, this is the first pedigree with C6Q0 deficiency reported in Chinese and the first TCC deficiency reported in Hong Kong. In contrast, a high prevalence of C6 deficiency has been reported among African Americans.^12^, ^21^ In South Africa, where C6Q0 seems most prevalent, four common frameshift defects in the *C6* gene account for the vast majority of cases. It is likely that C6Q0 patients of different ethnic and geographic backgrounds carry distinct mutations, hence the significance in detecting these two mutations in our reported pedigree. We postulate that these two mutations probably originated from distinct geographic regions as the parents of this family had originated from different prefectures of Guangdong in China: the father (I.1, inferred heterozygous for p.Arg596Ter) was from Shantou, while the mother (I.2, heterozygous for p.Arg606Ter) was from Shanwei. It would be of great interest to investigate the frequency of these C6 mutations in different areas of China and for potential founder's effect such as that seen in C9 deficiency in Orientals.^17^

Individuals with TCC deficiencies are well known to be susceptible to meningococcal disease with up to 1000‐ to 10 000‐fold increased risk compared with general population. Meningitis has been reported to occur in approximately 40% of individuals with TCC deficiencies.^22^ Interestingly, out of the three individuals we diagnosed with C6Q0, only the index patient had a history of recurrent meningococcal infections. We postulate other external factors, such as the index patient's smoking habits, may have further increased his susceptibility to infection. Cigarette smoking has been linked with increased risk of nasal carriage of *Neisseria meningitidis* and invasive meningococcal infections, presumably via increased expression of adhesion molecules in the nasopharynx and alterations of the local cytokine milieu which facilitates bacterial invasion.^23^, ^24^ In addition to environmental factors, other components of the immune system may also play a role in further modulating the risk of *Neisseria* infections among already‐susceptible individuals. For example, variations in the Fcγ receptor family in humans have also been reported to increase the risk of meningococcal infections.^25^, ^26^ Such multifactorial differences may explain the heterogeneous clinical presentations among C6Q0 individuals. It would be of great interest to further investigate for further possible environmental and immunological susceptibility factors among C6Q0 pedigrees in future studies.

In general, complement deficiencies remain one of the rarer causes of PIDs and make up to < 10% of all PIDs in most national registries.^27^ Despite the dire consequences of missed diagnoses, physicians are often unaware of the possibility of PID. For example, the index patient had suffered at least three separate episodes of life‐threatening invasive *Neisseria* infections, complicated by permanent hearing loss, prior to immunologist referral. Furthermore, *Neisseria meningitidis* serotype Y less frequently causes infections in healthy individuals and should further prompt consideration of an immunodeficiency.^1^ Given that there is only one public immunology centre for adult patients in Hong Kong and scarcity of clinical immunologists in China, many PIDs including complement deficiencies are likely to be under‐estimated and under‐reported in Chinese. Access to comprehensive immunological investigations, such as searching or testing for complement deficiencies, is extremely limited for Chinese immunologists and will be a priority for future development.

In the past, when various molecular diagnostic platforms were not available, such cases required protracted investigations by assaying individual components of the TCC. Individual complement components had to be measured one by one by ELISA or their functions assessed with various component‐depleted sera, which are seldom readily available. There are also marked variations in assay performance, lack for standardisation between different centres and difficulties in sample handling because of the labile nature of complement proteins. These assays are therefore rarely requested and difficult to validate for routine practice in comparison with the NGS technology. With NGS, multiple genes of interest can be analysed simultaneously. For example, in this case, multiple genes of interest (i.e. C5 to C9) were all investigated in a single assay run. Sample collection is also much easier since DNA is a stable analyte and only a small amount of blood required. The assay set‐up can be done in molecular laboratories with the sequencing platform, and there are relatively standardised technique and assay procedures. Although variants still need to be properly assessed for their functional and disease relevance, NGS has proven to be a powerful tool to facilitate and expedite patient workup and management.

There are several potential limitations to our study. Although the C6 level was measured, further functional studies on the effect of normal serum versus known C6‐depleted serum in correcting the complement haemolytic activity have not been performed. Furthermore, complement haemolytic activity is unstable and heat‐labile, and therefore, all haemolytic assays were repeated for confirmation of any abnormal findings. Also, because of the detection limit of the ELISA, it was not possible to differentiate between total absence and near absence of the C6 protein.

In conclusion, we report on two compound heterozygous mutations in *C6*, p.Arg596Ter and p.Arg606Ter inherited in three patients of the first recorded Chinese pedigree of C6Q0. Neither mutations had been reported to be pathogenic previously. We demonstrate through extensive serological and genetic testing that these single heterozygotes develop C6SD while compound heterozygotes develop C6Q0 with classical predisposition to recurrent meningococcal disease. Based on the family history, we postulate that these mutations originated from distinct geographic regions in China. It is likely that the genetic causes of C6Q0 (and other PIDs) in Chinese may vary significantly compared with other populations and highlights the importance of future immunology studies dedicated to specific ethnicities.

## Methods

### Index case and clinical vignette

A 22‐year‐old Chinese man was first admitted to his local hospital for fever, neck stiffness and headache which did not respond to an initial course of oral antibiotics. Computerised tomography (CT) brain was unremarkable, and a lumbar puncture was diagnostic for acute bacterial meningitis. Cerebrospinal fluid (CSF) and blood cultures and microbiological cultures were negative, likely because of prior antibiotic use. He responded to a course of intravenous ceftriaxone.

One year later, he presented again with similar symptoms but complicated by septic shock. Blood and CSF cultures yielded *Neisseria meningitidis* serotype Y. He responded to a course of ceftriaxone but was complicated by residual left‐sided hearing deficit. Six months thereafter, he was admitted again for a third episode of meningitis. Both blood and CSF cultures yielded *Neisseria meningitidis* serotype Y and again responded to a course of ceftriaxone. CT and magnetic resonance imaging of the brain, sinuses and base of skull were unremarkable. Review by his neurologist and otorhinolaryngologist did not identify any structural abnormalities or cause for recurrent meningitis. He was then referred to immunology clinic for investigation.

The patient was a smoker and social drinker, working as a salesperson. He was born and raised in Hong Kong without significant past medical history. There was no history of frequent infections, and he received all vaccinations according to the local immunisation programme without problems.^28^ He had no history of sexually transmitted diseases, including *Neisseria gonorrhoeae*.

Complete blood count and differentials; liver and renal function tests; immunoglobulin levels and lymphocyte subsets (including CD3^+^, CD8^+^, CD19^+^, CD16^+^/56^+^) were unremarkable. Antihepatitis B surface and anti‐A/‐B isohemagglutinins were present. Human immunodeficiency virus antibody/antigen was negative. Repeat measurements of both CH50 and AH50 complement assays showed absent haemolytic activity, along with normal serum C3 level (95 mg dL^−1^; reference range 76–150 mg dL^−1^), suggesting a TCC deficiency. NGS with targeted enrichment of C5 to C9 was employed for detection of genetic defects, as well as C6 ELISA was performed, as described below.

### Family screening

The patient was the youngest of six siblings of the same nonconsanguineous parents (Figure 2). Both parents and six siblings were asymptomatic and received all vaccinations according to the local immunisation programme without problems. There was no family history of recurrent infections or meningococcal disease. The patient's father (I.1) died of a traffic accident. His second sibling (II.2) was unavailable for testing because of social reasons. All other family members consented to serological and genetic testing.

### Complement haemolysis assays (CH50/AH50)

Both the classical and alternative functional pathways were measured by performing both CH50 and AH 50 assays. The CH50 assay assessed the ability of serum complement factors C1‐C9 to lyse sheep erythrocytes, while the AH50 assessed complement factors B, D, P and C3‐C9 involved in alternative pathway to lyse rabbit erythrocytes. Results were expressed as the reciprocal of the dilution giving 50% lysis (CH50 units mL^−1^). The CH50/AH50 assays employed were developed in‐house by the Clinical Immunology Laboratory of Queen Mary Hospital, Hong Kong.

### Genomic DNA sequencing and quantification of serum C6 levels

Peripheral blood samples were collected from the index patient and his available family members. For the indexed patient; NGS was deployed for sequencing *C5* to *C9* because of its efficiency to analyse a large number of exons simultaneously. The commercial TruSight One capture panel (Illumina, San Diego, CA, USA) was used for sequencing the entire gene regions of *C5* to *C9* genes. Sample enrichment and paired‐end libraries were generated according to the manufacturer's instructions. Sequencing of the generated library was carried out on the Illumina NovaSeq 6000 platform (Illumina). Variant calling was performed using the BaseSpace Onsite Sequence Hub. Sanger sequencing was used to confirm the variant(s) detected in the indexed patients or for subsequent detection of variant(s) in the other family members (PCR primers and conditions are detailed in Table 2).

**Table 2:** Primers and conditions used for Sanger sequencing

Primer name^a^	Direction	Primer sequence (5′ ‐> 3′)^b^	Product size	Targeted location
C6‐E12F	Forward	TTCAGCTGCACCATGATCCAT	345	Exon 12
C6‐E12R	Reverse	TCTGTGTTGGCATAGGTAAAGT		

^a^ PCR using the forward primers and reverse primers was performed in a 25‐μL total reaction volume: GoTaq^®^ G2 Hot Start Polymerase kit (Promega, WI, USA) containing 5 μL of ColorLess GoTaq^®^ Flexi Buffer (Promega, WI, USA), 2.5 μL of 25 mm Magnesium Chloride (Promega, WI, USA), 0.5 μL of 10 mm PCR nucleotide mix (Roche, Basel, Switzerland), 0.5 μL of each primer (10 μm), 10 μL of DNA extract (final conc. 100 ng), 0.125 μL (5 units μL^−1^) of GoTaq^®^ G2 Hot Start Polymerase, and 5.875 μL of deionised water. Thermal cycling conditions were as follows: 95°C hold for 5 min; 35 cycles of 95°C for 30 s, 57°C for 30 s and 72°C for 90 s; and a 72°C hold for 7 min. A negative water control and a wild‐type control were included for quality control purposes.

^b^ Primer sequences from Hobart *et al*.^29^

The C6 ELISA assay (Abcam, Cambridge, UK) was employed to quantitate the level of C6 in the index patient and his family members. Results are expressed in ng mL^−1^.

All subjects gave their informed consent for all molecular and genetic testing.

## Conflict of interest

The authors declare no conflict of interest.

## Author contributions

**Philip H Li:** Conceptualization; Data curation; Formal analysis; Investigation; Methodology; Project administration; Resources; Software; Supervision; Validation; Visualization; Writing‐original draft; Writing‐review & editing. **William WY Wong:** Conceptualization; Data curation; Formal analysis; Investigation; Methodology; Visualization; Writing‐original draft. **Evelyn NY Leung:** Formal analysis; Investigation; Methodology; Writing‐review & editing. **Chak‐sing Lau:** Conceptualization; Project administration; Resources; Supervision; Validation; Visualization; Writing‐review & editing. **Elaine Au:** Conceptualization; Formal analysis; Investigation; Methodology; Project administration; Validation; Writing‐original draft.
